# Exploring Pseudoainhum in Camisa syndrome

**DOI:** 10.1002/ccr3.4995

**Published:** 2021-10-23

**Authors:** Bibisha Baaniya, Sudha Agrawal

**Affiliations:** ^1^ Junior Resident B.P. Koirala Institute of Health Sciences Dharan Nepal; ^2^ Department of Dermatology and Venerology B.P. Koirala Institute of Health Sciences Dharan Nepal

**Keywords:** acitretin, camisa syndrome, pseudoainhum

## Abstract

Immediate treatment of Camisa syndrome with systemic retinoids or surgery helps to prevent loss of digits. Here, we report a case of Camisa syndrome with pseudoainhum in the fifth toe leading to amputation as timely treatment was not sought.

## Graphical abstract

Timely recognition and prompt management of Camisa syndrome are essential to prevent the pseudoainhum formation and thus amputation.

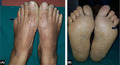

## 1 INTRODUCTION

Vohwinkel syndrome (VS), also known as keratoderma hereditaria mutilans, is a rare, autosomal dominant, and syndromic form of diffuse palmoplantar keratoderma (PPK) which manifests as hyperkeratosis of the palms and soles with a honeycomb appearance.^1^

Vohwinkel's syndrome is classified into two variants: (1) a deafness‐associated variant (Classical) and (2) an ichthyosis‐associated variant (Camisa syndrome).^2^ Camisa syndrome, also called as variant Vohwinkel's syndrome or loricrin keratoderma, is a rare variant that is associated with ichthyosis most commonly ichthyosis vulgaris and lamellar ichthyosis.^3^ Pseudoainhum (constricting circumferential band around a digit or limb) is one of the classical features of this syndrome while starfish‐shaped keratotic papules and deafness are not observed.^4^, ^5^

Recently, it has been shown that gain‐of‐function mutations in LOR on 1q21.3. underlies the ichthyotic variant while that in connexin 26, genes causes VS with deafness.^6^, ^7^ The histological features of Camisa syndrome include hyperkeratosis with orthokeratosis and focal parakeratosis, acanthosis, elongation of rete ridges, and sparse dermal lymphocytic infiltrate with normal appendages.^8^, ^9^ Retinoids such as acitretin and isotretinoin have been prove to be effective in hereditary palmoplantar keratoderma and preventing pseudoainhum.^10^, ^11^

Here, we report a case of Camisa syndrome, who lost her digit due to lack of timely treatment.

## 2 CASE REPORT

**FIGURE 1:**
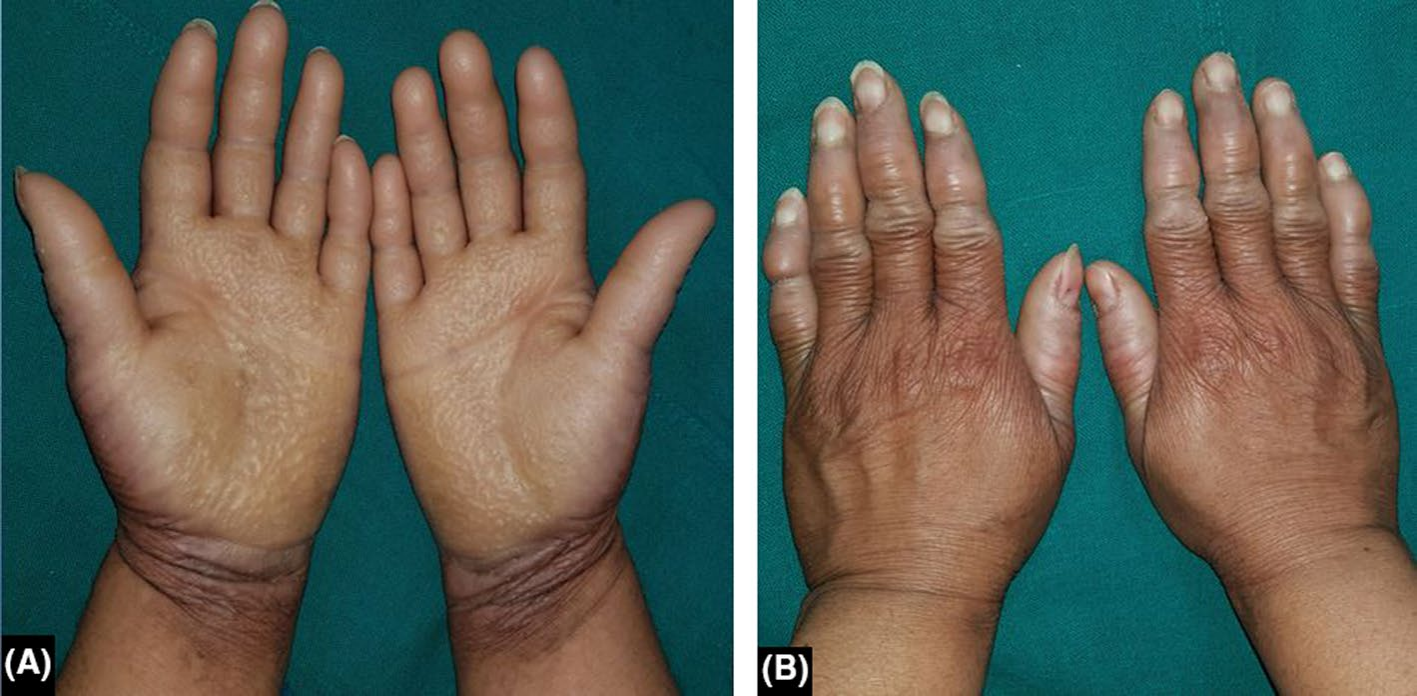
Brownish honeycomb transgradient hyperkeratosis in bilateral palms and circumferential constriction in eight fingers

**FIGURE 2:**
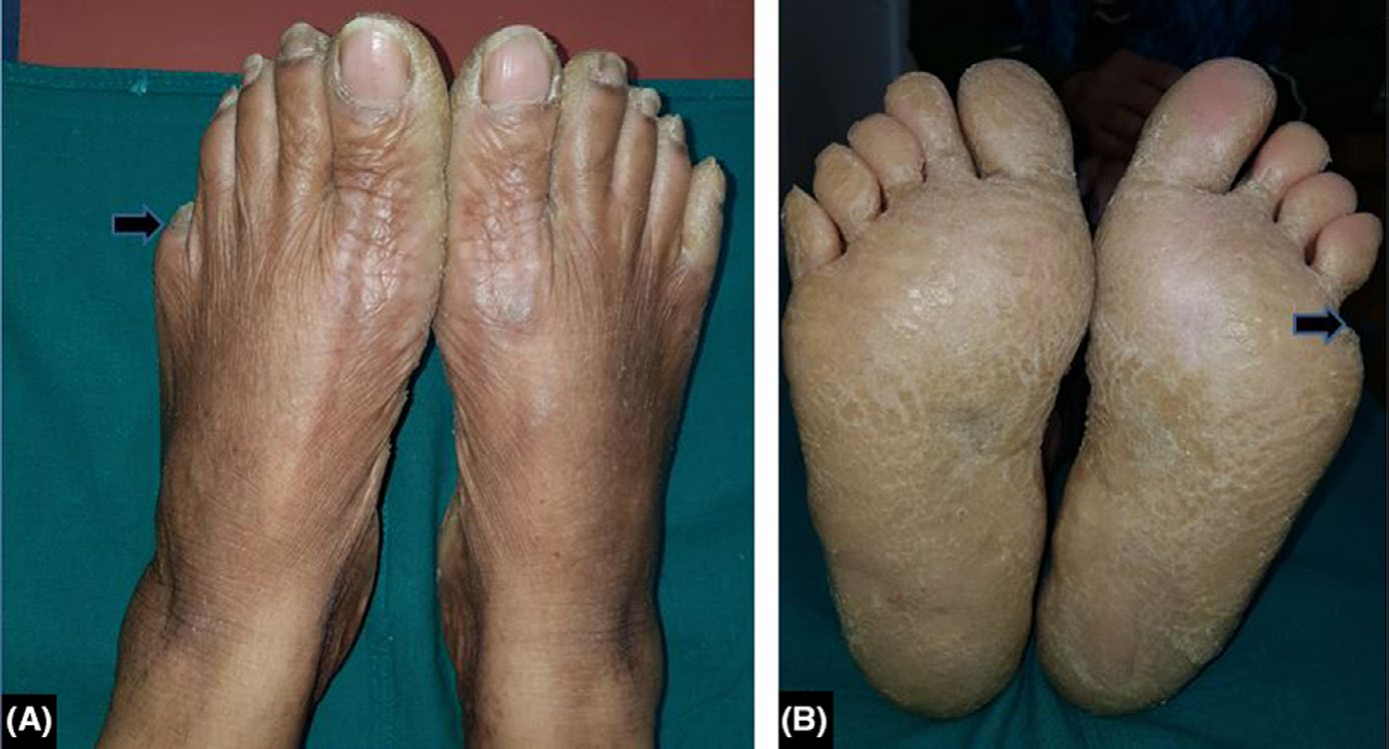
Pseudoainhum with amputation at the level of proximal interphalangeal joint (arrow heads)

We present a case of 38‐year‐old woman who presented to our Dermatology OPD with complaints of palmoplantar thickening and scaling. During her childhood, she had noticed slight scaling localized to bilateral palms and soles which then progressed to become brownish honeycomb transgradient hyperkeratosis over a period of around ten years (Figure 1A, B; Figure 2A, B). It was associated with pain, tightening, and winter aggravation. She also had generalized dryness of the skin. However, she did not receive any treatment, and at the age of twenty‐five years, she gradually developed a constriction band around the fifth toe which gradually tightened leading to pseudoainhum with amputation at the level of the proximal interphalangeal joint (Figure 2A, B). Her hearing, vision, hair, and nails were normal. Among her two children, the elder son is eleven years old and is also affected and the younger daughter is unaffected till this date. Considering the presence of transgradient honeycomb keratoderma with generalized ichthyosis and pseudoainhum in the absence of deafness, a diagnosis of Camisa syndrome was made.

## 3 TREATMENT AND FOLLOW‐UP

Initially, she was treated with oral acitretin, and currently, she is under maintenance therapy with topical urea and salicylic acid. She is also being closely followed up to assess for further progression of disease and formation of pseudoainhum.

## 4 DISCUSSION

Vohwinkel syndrome was first described in 1929 by Vohwinkel. VS with ichthyosis (Camisa's syndrome) is a rare group of inherited genodermatoses, with very few cases reported in the literature.^1^ It has an autosomal dominant inheritance due to mutations in LOR, starting in childhood, and occurring predominantly in females and Caucasians.^7^, ^12^ Our case and her son also had childhood onset. Furthermore, it can be made out that the mother had sporadic condition and the son got it from her in an autosomal dominant fashion.

Clinical features of Camisa's syndrome include generalized ichthyosis and honeycomb‐like palmoplantar keratoderma, with or without varying degrees of constricted digits, erythematous plaques, and/or erythroderma.^7^, ^8^ Honey comb‐like keratoderma, pseudoainhum, and ichthyosis were present in our patient, and her hearing was normal. Pseudoainhum and amputation are most common in the fifth toe, which was the case in our patient as well. (Table 1).

**TABLE 1:** Case reports of Camisa disease

SN	Authors	Country	No. of cases	Cases with Pseudoainhum	Digits	Age of pseudoainhum	Amputation	Treatment
1	Kura, Parsewar^13^	India	1	1	Left 5th toe	14 years	Absent	Acitretin 25 mg PO BD x 6 months
2	Reinehret al^5^	Brazil	19 (1 family)	1	2nd and 5th finger of hand	Not mentioned	Absent	Not mentioned
3	O’Driscoll et al^2^	UK	14 (1 family)	1	5th toes of bilateral feet	Not mentioned	Left 5th toe at 26 years and right 5th toe at 44 years	Surgical amputation done
4	Korge et al^8^	Scotland	8 (1 family)	Present but number not specified	Not mentioned	Not mentioned	One had amputation	Not mentioned
5	Armstrong et al^16^	UK	8 (1 family)	6	Not mentioned	Not mentioned	Absent	Not mentioned
6	Maestrini et al^17^ Schmuth et al^18^	USA	16 (1 family)	Nearly all (number not specified)	4th or 5th digit of hands or feet	Not mentioned	Some had amputation of bilateral 5th toe	Not mentioned
7	Rajashekar et al^9^	India	1	1	Right 1st and 5thtoes	Not mentioned	Absent	Not mentioned
8	Corte et al^19^	Brazil	2 (1 family)	2	Left 5th toe	Not mentioned	Present in 1 patient	Not mentioned
9	Camisa, Rossano^3^	Not available	Not available	Absent	Not applicable	Not applicable	Not applicable	Isotretinoin
10	Takahashi et al^20^	Japan	1	1	All fingers and 5th toes	Not mentioned	Absent	Not mentioned
11	Zamiri et al^15^	UK	1	1	All fingers	Not mentioned	Absent	Acitretin, keratolytic, full‐thickness skin graft
12	Suzuki et al^21^	Japan	8 (2 families)	5	Not mentioned	Not mentioned	Not mentioned	Not mentioned
13	Nico, Fernandes^10^	Brazil	1	1	Right 5th toe	Not mentioned	Right 5th toe at 25 years	Isotretinoin 0.5 mg/kg x 8 months
14	Hotz et al^22^	Brazil, Brazilian origin, France	4 (3 families)	3	1st—mild constriction 2nd—Fingers 3rd – 5th toe	Not mentioned	Absent	Not mentioned
15	Kinsler et al^23^	Not specified	5 (1 family)	Not mentioned	Not applicable	Not applicable	Not applicable	Not mentioned
16	Mu~noz‐Aceituno et al^24^	Spain	2 (1 family)	Absent	Not applicable	Not applicable	Not applicable	Emollients
17	Matsumoto et al^25^	Japan	3 (1 family)	3	PIP of all fingers	1st—3 years Not mentioned in the other 2 cases	Absent	Not mentioned
18	Gedicke et al^26^	Germany	5 (1 family)	Absent	Not applicable	Not applicable	Not applicable	Not mentioned
19	Drera et al^27^	Italy	2 (1 family)	1	DIP and PIP of all fingers	Not mentioned	Absent	Surgical treatment of pseudoainhum
20	Yeh et al^28^	Taiwan	3 (1 family)	3	DIP of all fingers	Early childhood	Absent	Not mentioned
21	Song et al^29^	China	15 (1 family)	Absent	Not applicable	Not applicable	Not applicable	Not mentioned
22	Pohler et al^30^	Not specified	10 (1 family)	Absent	Not applicable	Not applicable	Not applicable	Not mentioned
23	Khalil et al^31^	Iraq	1	1	Not specified	Not mentioned	Absent	Not mentioned
24	Ishida‐Yamamoto et al^32^	Japan	5	2	All fingers	Not mentioned	Absent	Not mentioned
25	Present case	Nepal	2	1	Left 5t^h^ toe, 2nd to 5th fingers of both hands	25 years	30 years	Acitretin, urea, salicylic acid

Here, we list the case reports and series of Camisa syndrome and associated pseudoainhum. (Table 1).

Acitretin is a feasible, conservative, and durable therapeutic modality for the treatment of pseudoainhum‑like mutilations associated with ichthyoses and mutilating keratodermas.^13^ The use of isotretinoin in a non‐continuous regimen is a reasonable approach in women of childbearing age, especially in cases at risk of amputation due to severe pseudoainhum.^10^ For pseudoainhum threatening, the viability of the digits excision of the constricting band followed by Z‐plasty has been reported to be effective.^14^ Successful full‐thickness skin grafting for pseudoainhum has also been reported.^15^ Our patient was treated with oral acitretin and topical urea and salicylic acid. This has halted the further progression of disease and loss of other digits.

This case highlights the importance of recognition and prompt treatment of Camisa syndrome to prevent the pseudoainhum formation and improve the quality of life.

## CONFLICTS OF INTEREST

The authors declare no conflict of interest.

## AUTHOR CONTRIBUTIONS

Bibisha Baaniya involved in preparation of manuscript and editing. Sudha Agrawal involved in Idea and literature review.

## 5 CONSENT

Patient provided written consent for publication of this case report.

## Data Availability

Data will be made available upon request.
